# Large Cell Neuroendocrine Carcinoma of the Rectum Presenting with Extensive Metastatic Disease

**DOI:** 10.1155/2014/386379

**Published:** 2014-07-16

**Authors:** Vinay Minocha, Sania Shuja, Robert Ali, Emely Eid

**Affiliations:** ^1^Division of Gastroenterology, Department of Internal Medicine, University of Florida, Jacksonville, FL, USA; ^2^Department of Pathology, University of Florida, Jacksonville, FL, USA

## Abstract

*Introduction*. Rectal large cell neuroendocrine carcinoma (LCNEC) is a poorly differentiated neoplasm that is very rare and belongs within the poorest prognostic subgroup among primary colorectal neoplasms. Here, we describe a case of LCNEC of the rectum, which highlights the aggressive clinical course and poor prognosis associated with this disease. *Case Presentation*. We report a case of a 63-year-old male who presented to our hospital with a one-month history of lower abdominal pain, constipation, and weight loss. A computed tomography (CT) scan of the chest, abdomen, and pelvis revealed a rectal mass as well as metastatic disease of the liver and lung. Flexible sigmoidoscopy revealed a fungating, ulcerated and partially obstructing rectal mass located 6 cm from the anal verge. This mass was biopsied and pathological examination of the resected specimen revealed features consistent with a large cell neuroendocrine carcinoma. *Conclusion*. Rectal large cell neuroendocrine carcinomas are rare and have a significantly worse prognosis than adenocarcinomas. At diagnosis, a higher stage and metastatic disease are likely to be found. It is important to differentiate large cell, poorly differentiated neuroendocrine carcinomas from adenocarcinomas of the colon and rectum pathologically because patients may benefit from alternative cytotoxic chemotherapeutic regimens.

## 1. Introduction

Neuroendocrine cells are located diffusely throughout the human body and are found in the gastrointestinal tract, pancreas, lung, thyroid, adrenal, and many other organs. Although the gastrointestinal tract has the largest proportion of neuroendocrine cells, neuroendocrine tumors (NETs) account for only 2% of all gastrointestinal malignancies [1]. These tumors are a heterogeneous group of neoplasms composed of cells containing neuroendocrine secretory granules in the cytoplasm which can be identified ultrastructurally. In the new WHO classification of 2010, neuroendocrine tumors are classified into well-differentiated and poorly differentiated. Well-differentiated tumors are G1 (low grade) or G2 (intermediate grade) NETs and poorly differentiated neuroendocrine carcinomas (NECs) are now called large cell type or small type G3 NECs [2, 3]. Both mitotic count and Ki-67 labeling index are considered for pathological classification into one of these three categories. NET G1 comprises well-differentiated neuroendocrine tumors that are low grade, with a low mitotic rate of less than 2 per 10 high-power fields (HPF) and a Ki-67 labeling index of <2%. NET G2 comprises well-differentiated neuroendocrine tumors that are intermediate grade, with a mitotic rate of 2–20 per 10 HPF and a Ki-67 labeling index of 3–20%. NET G3 are poorly differentiated neuroendocrine carcinomas, with a high mitotic rate of >20 per 10 HPF and a Ki-67 labeling index of >20%. Large cell or small cell neuroendocrine carcinomas fall within the NET G3 category. The majority of digestive tract neuroendocrine neoplasms are NET G1 also known as carcinoid tumors, with a relatively good prognosis [4]. Carcinoid tumors are usually solitary, less than 1-2 cm in size, and clinically indolent. By comparison, poorly differentiated NET G3 of the colon and rectum are rare tumors with a reported incidence of between 0.1% and 3.9% of all colorectal malignancies. These tumors are rare but very aggressive with most patients developing metastatic disease at an early stage of disease [5]. We describe a case of large cell neuroendocrine carcinoma (LCNEC) of the rectum, which highlights the aggressive clinical course and poor prognosis associated with this disease.

## 2. Case Report

A 63-year-old male presented to our hospital with a 1-month history of lower abdominal pain, constipation, and weight loss. He described the lower abdominal pain as cramping in nature with increased intensity during defecation and he claimed that his stool had decreased in caliber. He also experienced intermittent rectal bleeding and had an unintentional weight loss of 30 lbs during the past month. Physical examination was remarkable only for hepatomegaly on abdominal palpation. Lab investigations revealed an elevated white cell count of 15.5 × 10^3^/mm^3^ with a normal hemoglobin level of 14.7 g/dL and a normal platelet count of 319 × 10^3^/mm^3^. The patient also had elevated liver function tests with an alanine aminotransferase (ALT): 177 U/L, an aspartate aminotransferase (AST): 137 U/L, and an alkaline phosphatase: 518 U/L. Chest and abdominal radiographs were normal.

**Figure 1:**
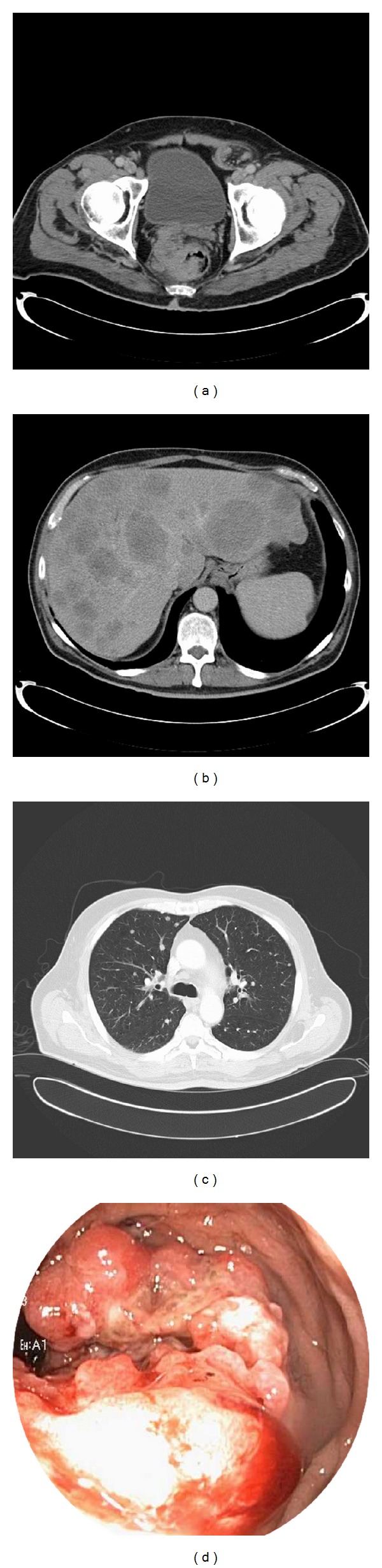
(a) Contrast-enhanced computed tomography scan of the pelvis reveals a rectal mass involving the anterior, right lateral, and posterior walls of the rectum. (b) Computed tomography scan showing numerous hypodense masses throughout the liver consistent with liver metastases. (c) Contrast-enhanced computed tomography scan of the chest showing bilateral lung nodules and mediastinal lymphadenopathy secondary to metastatic disease. (d) Picture from sigmoidoscopy showing a fungating, ulcerated, and partially obstructing rectal mass, involving two-thirds of the luminal circumference.

A computed tomography (CT) scan of the abdomen and pelvis revealed a rectal mass involving the anterior, right lateral, and posterior walls of the rectum beginning approximately 6 cm from the anal verge (Figure 1(a)). In addition, innumerable hypodense masses throughout the liver as well as retroperitoneal and pelvic lymphadenopathy were seen (Figure 1(b)). A subsequent CT chest showed numerous bilateral lung nodules and mediastinal lymphadenopathy (Figure 1(c)). These radiographic findings were consistent with widespread metastatic disease involving the liver and lung from a primary rectal cancer. Tumor markers were significant for an elevated CA19-9 of 178.7 U/mL and a normal carcinoembryonic antigen (CEA) and alpha-fetoprotein (AFP).

Flexible sigmoidoscopy revealed a fungating, ulcerated and partially obstructing rectal mass, involving two-thirds of the luminal circumference, located 6 cm from the anal verge (Figure 1(d)). This mass was biopsied and pathological examination of the resected specimen revealed features consistent with a large cell neuroendocrine carcinoma.

**Figure 2:**
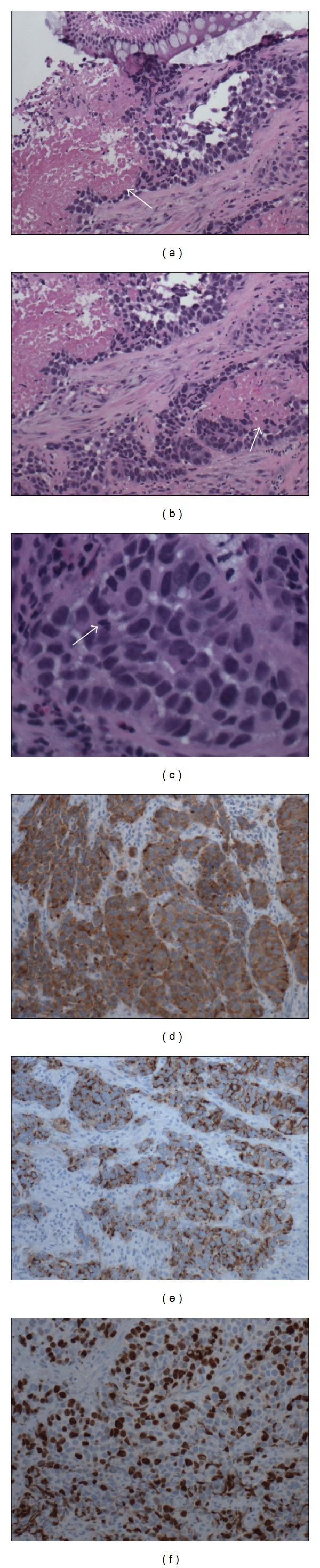
((a) and (b)) Large cell neuroendocrine carcinoma infiltrating the connective tissue stroma of the colorectal biopsy, as nests of cells. There is associated tumor necrosis (arrows). Normal colonic mucosa is visible at the top of the field in (a). (c) The neoplastic cells reveal hyperchromatic nuclei with inconspicuous nucleoli, and a mitotic figure (arrow) is visible to the left, in the nest of neoplastic cells. ((d), (e), and (f)) On immunohistochemical staining the neoplastic cells stained positive for synaptophysin (diffuse cytoplasmic brown staining), chromogranin (focal cytoplasmic brown staining), and expressing a high Ki-67 labeling index of up to 50% cells (brown nuclear staining).

On microscopic examination, nests of tumor cells infiltrated colonic stroma with associated tumor necrosis (Figure 2, A&B). The individual tumor cells exhibited moderate eosinophilic cytoplasm, hyperchromatic, pleomorphic nuclei and frequent mitotic figures (Figure 2(c)). Immunohistochemical study revealed cytoplasmic staining for neuroendocrine markers synaptophysin and chromogranin consistent with neuroendocrine differentiation (Figures 2(d) and 2(e)). Staining for proliferation marker Ki-67 revealed positive nuclear staining in up to 50% of cell nuclei consistent with a high proliferation rate in tumor cells (Figure 2(f)). The clinical and histopathological findings confirmed a diagnosis of large cell neuroendocrine carcinoma of the rectum.

Interestingly, subsequent investigations revealed that the patient had elevated urinary 5-hydroxyindoleacetic acid (5-HIAA) levels at 100.2 mg/24 hr in a 24-hour urine sample without any clinical manifestations of the carcinoid syndrome. The patient also displayed an elevated serum chromogranin A (CgA) level of 15 nmol/L in keeping with the presence of an underlying neuroendocrine tumor.

Due to the aggressive nature of the tumor as evident from its rapid progression and extensive metastases to the lung and liver, the medical oncology team decided to initiate the patient on a chemotherapy regimen of cisplatin and etoposide. After receiving one cycle of chemotherapy, the patient's clinical status deteriorated. One month after initial diagnosis, he developed multiorgan failure with progression of the underlying metastatic carcinoma. The patient died while being under hospice care.

## 3. Discussion

Colorectal large cell neuroendocrine carcinoma (LCNEC) is a poorly differentiated neoplasm that is very rare and belongs within the poorest prognostic subgroup among primary colorectal neoplasms [6]. In a study of 6,495 patients with colorectal cancer by Bernick et al., only 0.6% had neuroendocrine carcinomas, and only 0.2% had LCNECs [5]. Although these tumors are rare, it is important to differentiate neuroendocrine carcinomas from adenocarcinomas of the colon and rectum pathologically because patients may benefit from alternative cytotoxic chemotherapeutic regimens. The presented case highlights the typically aggressive clinical course of this disease with a tendency for metastasis at initial presentation.

As demonstrated in this case, the clinical presentation of colorectal LCNECs may not be different from that of conventional colonic adenocarcinomas. At diagnosis, however, a higher stage and metastatic disease are likely to be found. Patients with colorectal LCNEC usually present with abdominal pain, hematochezia, constipation, or tenesmus. Symptoms associated with excessive hormone production such as paraneoplastic and carcinoid syndromes may rarely be a presenting feature [7]. Our patient presented with symptoms related to a partially obstructing rectal mass. He showed no clinical manifestations of hormone hypersecretion despite having elevated 24-hour urinary 5-hydroxyindoleacetic acid (5HIAA) and elevated serum chromogranin A (CgA) levels. These findings are consistent with a study of pulmonary and extrapulmonary LCNEC by Faggiano et al., which revealed that the first symptoms of the disease were related to tumor burden in 73% of the patients while an increase in at least one biologic marker such as serum CgA and 24-hour urinary 5HIAA was observed in 93% of patients [8].

A proposed explanation for this interesting finding may stem from a study of neuroendocrine cancers of the colon and rectum by Saclarides et al., in which none of the patients displayed clinical manifestations of the carcinoid syndrome. Based on this finding, the authors of the study concluded that although neuroendocrine differentiation and hormone secretion occur at the cellular level, these tumors may produce biologically active compounds in amounts that are insufficient to produce a target organ response. Another possible explanation is that the peptides or amines secreted by these tumors may be rapidly broken down in the peripheral circulation [9].

Histologically, colorectal LCNEC resembles its well-described counterpart in the lung. The histological classification initially proposed by Travis et al., for LCNEC of the lung, was subsequently adopted by the World Health Organization for classification of these tumors of the digestive system [10]. Colorectal LCNEC is defined histologically by a neuroendocrine appearance under light microscopy with round or polygonal cells arranged in organoid, nesting, trabecular, rosette-like, and palisading patterns, frequently with large patches of geographical necrosis. These cells have ample cytoplasm, coarse chromatin, and frequent nucleoli. A high mitotic rate of 20 or greater per 10 HPF and/or Ki-67 proliferation index of >20% is seen. Additionally, these tumors usually stain positively for one or more neuroendocrine immunohistochemical markers such as chromogranin A, synaptophysin, neuron-specific enolase, and CD56 [6, 11].

The tumor in this case met the pathological criteria for diagnosis of a rectal LCNEC given the large cell morphology with a high number of mitoses, multifocal necrosis, and presence of neuroendocrine immunohistochemical features including diffuse cytoplasmic staining for synaptophysin and chromogranin.

Although our case showed no immunoreactivity for CK20, there have been several case reports of positive staining for CK20 in LCNEC of the colon and rectum. CK20 is a tumor marker that is traditionally confined to the intestinal epithelial, urothelial, and Merkel cells and positive staining for this marker in colorectal LCNEC may suggest a potentially common precursor for these tumors and colorectal adenocarcinoma [11].

The Ki-67 index is probably the best available marker of tumor cell proliferation. The Ki-67 antigen is detected in the nucleus of actively cycling cells and is strictly related to cell replication and not to DNA repair. Its role in predicting the invasive potential of gastrointestinal neuroendocrine tumors has been established. High proliferating tumors that have a Ki-67 greater than 10% have extensive angioinvasion and show a great potential to develop metastatic disease [1]. In the case presented, up to 50% of the tumor cell nuclei stained positive for Ki-67 in keeping with the aggressive nature of the tumor and widespread metastatic spread.

Survival rates for neuroendocrine carcinomas of the colorectum, as described by different authors, show that these tumors are very aggressive and are associated with a poor prognosis. As shown in this case, most patients tend to present at an advanced stage with metastatic disease. Similar to colonic adenocarcinoma, the liver is the most common site of metastasis for this tumor [7]. Based on various studies, the median survival rate for patients with neuroendocrine carcinomas of the colorectum is between 5 and 11 months with one-year survival rates of between 10 percent and 15 percent. In a study by Bernick et al., patients with colorectal LCNEC had an overall median survival of 10.4 months (range from 0 to 263.7 months) [5].

These gastrointestinal neuroendocrine carcinomas are morphologically and phenotypically related to pulmonary NECs (large and small cell types) and therefore cytoreductive chemotherapy is generally recommended for these tumors [3, 12]. LCNEC is managed primarily with platinum-based chemotherapy such as cisplatin/etoposide and cisplatin/irinotecan. This data is extrapolated from published data on the treatment of high-grade small cell carcinoma of the lung [12]. In our case, the decision to start a chemotherapeutic regimen of cisplatin and etoposide was based on National Comprehensive Cancer Network (NCCN) guidelines for small cell lung cancer. These guidelines recommend systemic chemotherapy with a platinum-based combination regimen for extensive stage disease [13]. Interestingly, a retrospective review conducted by Smith et al found that surgery, particularly in the presence of metastatic disease, does not offer a survival benefit for the majority of patients with neuroendocrine carcinomas of the colon and rectum [12]. Although more studies are required to evaluate the role of adjuvant chemotherapy and the optimal choice of chemotherapeutic regimen, the current literature suggests that a combination of cisplatin and etoposide may offer a survival benefit for patients with stage III and IV colorectal LCNEC [5, 14].

## 4. Conclusion

Colorectal large cell neuroendocrine carcinomas are rare, comprising less than 1 percent of colon and rectal cancers. However, these tumors have a significantly worse prognosis than conventional adenocarcinomas because most patients have metastatic disease at the time of diagnosis. More research is needed to establish the optimal treatment modality and in particular the role of various adjuvant chemotherapy regimens at different stages of this disease.
